# Therapeutic potential of p-cymene in mitigating alcohol-induced damage in umbilical cord-derived mesenchymal stem cells through restoration of Nanog, VEGF, and antioxidants levels; *in silico* and *in vitro* approaches

**DOI:** 10.1590/1414-431X2025e15185

**Published:** 2026-03-02

**Authors:** N. Aziz, T. Maqbool, M. Arooj, H.S. Afzal, A. Altaf, M. Atif, S. Naz, M.N.H. Malik

**Affiliations:** 1 Institute of Molecular Biology and Biotechnology/Center for Research in Molecular Medicine, University of Lahore, Lahore, Pakistan; 2 University College of Medicine and Dentistry, University of Lahore, Lahore, Pakistan; 3 Faculty of Health Science, Equator University of Science and Technology, Masaka, Uganda; 4 Department of Pharmacy, University of Lahore, Lahore, Pakistan; 5 Department of Physical Therapy, Allied Health Sciences, University of Lahore, Lahore, Pakistan

**Keywords:** UC-MSCs, p-Cymene, MTT, ELISA, Cellular injury

## Abstract

Human stem cells can divide and differentiate into various cell types. Umbilical cord-derived mesenchymal stem cells (UC-MSCs) are multipotent cells with high regenerative, anti-inflammatory, and immunomodulatory properties. Ethanol, the main component of alcoholic beverages, induces cytotoxicity and oxidative stress in cells. In recent years, natural compounds have gained attention for their potential protective effects against ethanol-induced cellular damage. p-Cymene is one such compound that acts as an antioxidant. This study aimed to evaluate the potential of p-cymene to reduce the harmful effects of alcohol on ethanol-induced cytotoxicity and oxidative stress in UC-MSCs through *in silico* and *in vitro* approaches. *In silico* pharmacokinetic and toxicity analyses of p-cymene were used, followed by *in vitro* evaluation using ethanol-injured UC-MSCs. Cell viability, antioxidant [glutathione (GSH), super-oxide dismutase (SOD)], inflammatory, proliferative, apoptotic, and wound healing assays were performed across different concentrations to assess protective effects. The pharmacokinetic analysis showed that p-cymene exhibited considerable pharmacokinetic properties by following Lipinski's Rule of Five. Toxicity analysis revealed no toxic effects of p-cymene, suggesting its potential as a natural compound. Further *in vitro* experimentation showed that p-cymene independently restored cell viability, reduced inflammation, stabilized Nanog, improved vascular endothelial growth factor (VEGF), enhanced antioxidants (GSH, SOD), promoted wound healing, and reduced cell death in ethanol-injured cells, with the 50 µM concentration being the most effective. These findings support our hypothesis that p-cymene protects UC-MSCs from ethanol-induced damage.

## Introduction

Human stem cells are an essential resource for regenerative medicine and tissue engineering, and any damage to these cells can have severe consequences. Human stem cells are specialized cells that have the unique ability to differentiate into many different types of cells in the body, including muscle, bone, blood, and nerve cells (1,2). Stem cells are essential for the development and maintenance of tissues and organs in the body and are a vital component of regenerative medicine (2). Therefore, investigating the protective effect of p-cymene against ethanol-induced cytotoxicity and oxidative stress in human stem cells is of great interest.

The excessive consumption of alcohol in various beverages can lead to various health issues, including damage to cells and tissues (3). Ethanol, the main component of alcoholic beverages, induces cytotoxicity and oxidative stress in cells, which can contribute to the development of various diseases (4). The detrimental effects of ethanol consumption on human health have been well-established, particularly in the context of the liver and other vital organs (5,6). Ethanol consumption has been identified as a major risk factor for a variety of diseases, including liver disease, cardiovascular disease, and cancer (7). One of the main mechanisms by which ethanol exerts its harmful effects is through the induction of oxidative stress, which can lead to cell death and tissue damage (4,8). Therefore, finding effective and safe protective agents against ethanol-induced toxicity is essential for maintaining the normal function of stem cells and preventing potential tissue damage.

Human stem cells, due to their proliferative and regenerative properties, are highly susceptible to ethanol-induced oxidative stress and cytotoxicity. Damage to these cells can compromise their therapeutic potential in regenerative medicine. Hence, identifying natural agents capable of preserving stromal cell function under ethanol-induced stress conditions is of critical importance. Given the established role of oxidative stress and inflammation in ethanol-mediated cellular injury, compounds with antioxidant and anti-inflammatory activities are promising candidates for protective intervention. Recent studies have highlighted the potential of natural compounds in shielding cells from damage induced by ethanol. One such compound is p-cymene, a monoterpene abundant in the essential oils of plants like cumin, thyme, and oregano. Research indicates that p-cymene possesses cytoprotective capabilities, defending cells against harmful stressors (9). A key mechanism behind its protective role is its antioxidant activity. By neutralizing free radicals (unstable molecules linked to oxidative stress), p-cymene helps prevent cellular damage associated with chronic conditions such as cancer, Alzheimer's disease, and Parkinson's disease (9). Additionally, p-cymene exhibits anti-inflammatory effects, mitigating inflammation-related cellular harm and supporting overall tissue protection (10).

Moreover, studies have suggested that p-cymene may have protective effects against various toxins and drugs, including chemotherapy drugs, by reducing their toxic effects on cells and tissues (11). Several studies have reported the beneficial effects of p-cymene against toxicity induced by various toxic agents. However, the protective effect of p-cymene against ethanol-induced toxicity in human stem cells has not been extensively studied (12).

In the current study, the protein targets tumor necrosis factor (TNF)-α, prostaglandin-endoperoxide synthase 2 (PTGS2), nuclear factor (NF)-κB, and matrix metalloproteinase (MMP)2 were selected for molecular docking analysis because they represent critical regulators of ethanol-induced cytotoxicity and inflammation. TNF-α and NF-κB are central mediators of pro-inflammatory signaling; PTGS2 (COX-2) is a key enzyme in oxidative stress and inflammatory cascades, while MMP2 contributes to extracellular matrix remodeling and tissue damage. These proteins were identified as hub targets in our network pharmacology analysis and, therefore, prioritized for *in silico* binding studies with p-cymene. In contrast, the *in vitro* experiments focused on downstream biomarkers (TNF-α, interleukin (IL)-6, transforming growth factor (TGF)-β1, p53, vascular endothelial growth factor (VEGF), and Nanog) to capture functional outcomes of ethanol toxicity and its modulation by p-cymene.

In this study, we aimed to investigate the protective effect of p-cymene against ethanol-induced cytotoxicity and oxidative stress in human stem cells. We hypothesized that p-cymene protects human stem cells from the harmful effects of ethanol and prevents ethanol-induced damage. The results of this study can provide valuable insights into the potential of p-cymene as a protective agent against ethanol-induced cellular damage and pave the way for the development of novel therapeutic strategies for alcohol-related diseases.

## Material and Methods

### Study setting

This experimental study was conducted at the Institute of Molecular Biology and Biotechnology University of Lahore after obtaining approval from the Institutional Review Board of the University of Lahore (Ref-IMBB/BBBC/23/213).

### Target identification and data collection

The drug targets of p-cymene were systematically identified using Swiss Target Prediction and PharmMapper, which predict potential protein interactions based on chemical structure and pharmacophore features (13). For disease-associated targets related to ethanol-induced toxicity, genes were retrieved from three major databases: Comparative Toxicogenomic Database (CTD) (14), DisGeNET, and Gene Cards. These databases provide comprehensive, experimentally validated gene-disease associations. Duplicate entries were removed to generate a refined list of unique targets for subsequent analysis (15).

### Identification of common targets

To determine the overlapping targets between p-cymene and ethanol-induced toxicity, a Venn diagram was constructed using Venny 2.1. This tool facilitated the visualization and extraction of shared genes, which represent potential therapeutic targets for further investigation. The intersection of drug and disease targets provided a focused set of genes likely to mediate the protective effects of p-cymene against ethanol-induced damage (16).

### Protein-protein interaction network construction

To elucidate the molecular mechanisms underlying the protective role of p-cymene against ethanol-induced injury in umbilical cord-derived mesenchymal stem cells (UC-MSCs), a protein-protein interaction (PPI) network was constructed based on overlapping gene targets. “Common targets” were defined as the intersection between p-cymene-associated targets and ethanol-injury-related targets, representing candidate genes that may mediate the protective effects of p-cymene. These common targets were then imported into the STRING database (version 11.5), with the organism set to *Homo sapiens* and the minimum required interaction score fixed at 0.7 (high confidence). Nodes without interactions were excluded to optimize network clarity. The resulting PPI network was visualized using Cytoscape (v. 3.9.1) to identify hub proteins (17).

### Hub gene selection

Key hub genes within the PPI network were identified using the CytoHubba plugin in Cytoscape. The Degree method was applied to rank nodes based on their connectivity, with the top 10 highest-degree genes selected as critical regulators. These hub genes are likely to play central roles in the biological mechanisms underlying ethanol toxicity and p-cymene's protective effects (18).

### Functional enrichment analysis

To elucidate the biological roles of the identified targets, Gene Ontology (GO) and Kyoto Encyclopedia of Genes and Genomes (KEGG) pathway analyses were performed using ShinyGO (v. 0.77) (19). This tool provided insights into enriched biological processes, molecular functions, cellular components, and signaling pathways. Statistical significance was set at a false discovery rate (FDR) <0.05, ensuring robust and reliable results (20).

### Molecular docking studies

Binding interactions between p-cymene and key target proteins TNF-α, PTGS2, NF-κB, and MMP2 were investigated using Molecular Operating Environment (MOE; v. 2024.6). Protein structures were obtained from the Protein Data Bank (PDB), prepared by removing water molecules and optimizing protonation states, and energy-minimized using the AMBER10: EHT force field. The 3D structure of p-cymene was similarly prepared for docking. Docking simulations were performed with the London dG scoring function, followed by refinement using GBVI/WSA dG. The top binding targets were selected based on binding affinity (S Score) and root-mean-square deviation (RMSD) values. Interaction analyses, including hydrogen bonds, hydrophobic contacts, and pi-stacking, were conducted to evaluate binding stability and specificity (21).

#### Isolation and culturing of cells

Isolation and culturing of cells were performed using enzymatic or mechanical methods to extract cells from tissues. Briefly, umbilical cord tissue was minced into small fragments and digested with collagenase type I and DNase I at 37°C for 1-2 h. The cell suspension was filtered, centrifuged (300 *g* for 5 minutes at 4°C), and resuspended in Dulbecco's Modified Eagle Medium (DMEM) supplemented with 10% fetal bovine serum (FBS), 1% penicillin-streptomycin, and essential growth factors. Cells were cultured in a humidified incubator at 37°C with 5% CO_2_, monitored regularly, and subcultured upon reaching 80-90% confluence to maintain viability (22).

#### Assessment of UC-MSCs with flow cytometry and morphology

The assessment of UC-MSCs using flow cytometry and morphology was conducted. Briefly, cells at passage 3 were harvested using trypsin-EDTA, washed with phosphate-buffered saline (PBS), and incubated with fluorophore-conjugated antibodies against a cluster of differentiation markers CD29, CD44, CD73, and CD90, while CD11b/c and CD45 were used as negative markers to confirm the mesenchymal stem cell phenotype and exclude hematopoietic lineage cells. Flow cytometry was performed using a BD FACSCanto II, and data were analyzed with FlowJo software to confirm the UC-MSC phenotype (23).

Morphological assessment was performed using an inverted phase-contrast microscope (Leica, Germany) to observe cell adherence, spindle-shaped fibroblast-like appearance, and colony formation.

### Viability analysis of p-cymene on umbilical cord stem cells

Cells were cultured in a 96-well plate for viability analysis. Briefly, the cells mixed in the culture media were plated onto a 96-well plate, allowing the cells to adhere to the plate. To maintain the cells in the plate, the medium was replaced every 2-3 days, allowing UC-MSC growth and removing non-adherent cells (24).

### Treatment

The cells were divided into 5 groups: control (untreated group), injury group (ethanolic group), treatment group (p-cymene 10-50 µM), and positive control group (Silymarin; Sigma-Aldrich, USA). When the cells reached 70-80% confluence, they were treated with 10% alcohol and varying concentrations of p-cymene (10, 25, and 50 µM) to determine the viable concentration. For the MTT assay, crystal violet staining, and trypan blue exclusion test, the cells were seeded onto a 96-well plate. After alcohol exposure, a 96-well plate was used to determine the effect of the maximum concentration of 50 µM of p-cymene. Remaining concentrations were exposed after 24 h on the plates. A 24-well plate was used for the scratch assay to evaluate cell migration and immunostaining. Additionally, the cell culture supernatant was collected and analyzed using ELISA to assess secreted factors.

### Cell viability assessment

The MTT assay was performed to assess cell viability. Cells were treated for 24 h with varying concentrations of alcohol as described above. Following this, they were rinsed with PBS and incubated with 100 µL of DMEM and 25 µL of MTT solution (Invitrogen Inc., USA) for 3-4 h. After a 4-h incubation period (37°C), 10% sodium dodecyl sulfate (SDS) (Invitrogen Inc.) was used to solubilize the formazan crystals, and an absorbance measurement was made at 570 nm. Percent viability was calculated according to the usual formula (12).

### Detection of living cells

The crystal violet assay was conducted on a 96-well plate. PBS was used to rinse the cells, and a mixture of crystal violet and ethanol with concentrations of 0.1 and 2%, respectively, was added to each well after covering the well. The mixture was kept for about 15 min at room temperature. Afterward, the content of the well was discarded and washed carefully to prevent the cells at the base from detaching from the well. Finally, 100 µL SDS (1%) was added to each well, and a microtiter plate was used to measure absorbance at 505 nm (25).

### Detection of dead cells

This assay was performed to evaluate the proportion of dead cells. After removing the culture medium, the wells were rinsed three times with PBS. The cells were then treated with trypan blue solution (Invitrogen Inc.) for 5 min to assess viability. Following incubation, the wells were washed again three times with PBS to remove excess dye. Microscopic examination was performed to evaluate cell staining, where cells retaining trypan blue uptake were considered as non-viable (18).

### ELISA

ELISA was performed to quantify various growth and inflammatory markers associated with wound healing (IL-6, TNF-α, TGF-β1, Nanog, p53, and VEGF). After treatment, the culture media from each experimental group were collected and centrifuged at 1,000 *g* for 10 min at 4°C to remove debris. The clear supernatant (cell culture medium) was used for all ELISA assays. Commercially available ELISA kits (Abcam, UK) were used according to the manufacturer's instructions. Briefly, 100 μL of standards and samples were added to the antibody-coated wells and incubated for 2 h at room temperature. After washing three times with the provided wash buffer, 100 μL of biotin-conjugated detection antibody was added to each well and incubated for 1 h. Wells were again washed, followed by the addition of 100 μL of HRP-streptavidin solution and incubation for 45 min. After the final wash, 100 μL of TMB substrate solution was added, and color development was allowed for 10-15 min in the dark. The reaction was stopped by adding 50 μL of stop solution, and the absorbance was measured at 450 nm using a microplate reader (Bio-Rad, PR4100, USA).

### Evaluation of antioxidant enzymes

#### Glutathione (GSH) assay

Each well of a 96-well plate had 200 μL of reaction mixture added for the GSH assay. KH2PO4 buffer (20 mM, pH 7.5), 40 mM of EDTA, and 10 mM of oxidized glutathione were mixed together to make the solution. Cell culture supernatant isolated from the treatment groups was added to the reaction mixture. The absorbance at 340 nm was then measured using ELISA Reader (Bio-Rad, PR4100), and a 20-mM NADPH solution was added to the mixture (26).

#### Superoxide dismutase (SOD) assay

In this study, a buffer solution comprising 100 mM KH_2_PO_4_ (pH 7.8), 0.1 mM EDTA, 13 mM methionine, 2.25 mM nitro-blue tetrazolium chloride (NBT), and 60 mM riboflavin was combined with the cell culture supernatant of the experimental groups. A spectrophotometer was used to determine the optical density at 560 nm (27).

#### Immunostaining with DAPI and PI

The cell samples were initially fixed using a 4% paraformaldehyde solution for 15 min under ambient temperature conditions. Subsequent permeabilization was performed with a 0.1% Triton X-100 solution for 10 min. To prevent nonspecific binding, samples were treated with 3% bovine serum albumin (BSA) blocking solution for 30 min. Nuclear visualization was achieved through 5-min staining with DAPI at a concentration of 0.5 µg/mL. For cell cycle analysis, simultaneous treatment with propidium iodide (PI, 10 µg/mL) and RNase A (100 µg/mL) was conducted for 30 min at room temperature. After thorough PBS washes, the prepared specimens were mounted and examined using fluorescence microscopy (Floid Cell Imaging Station, Life Technologies).

### Statistical analysis

Data was analyzed by one-way ANOVA followed by Tukey's *post hoc* test for multiple comparisons to make comparisons within the groups using Graphpad Prism 8.0.2. P-values <0.05 were considered statistically significant.

## Results

### Distribution of drug and disease targets in the dataset

**Figure 1:**
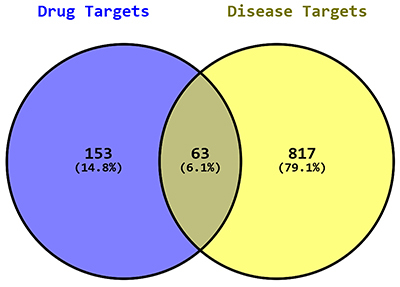
Venn diagram showing the distribution of drug targets (14.8%), disease targets (79.1%), and common targets (6.1%) in the analyzed dataset.

The Venn analysis revealed a total of 1,033 targets. Among these, 153 (14.8%) were identified as drug targets, while 63 targets (6.1%) were identified as common targets. The remaining 817 targets (79.1%) were categorized as disease targets (Figure 1).

### Protein-protein interaction network analysis and identification of hub genes

**Figure 2:**
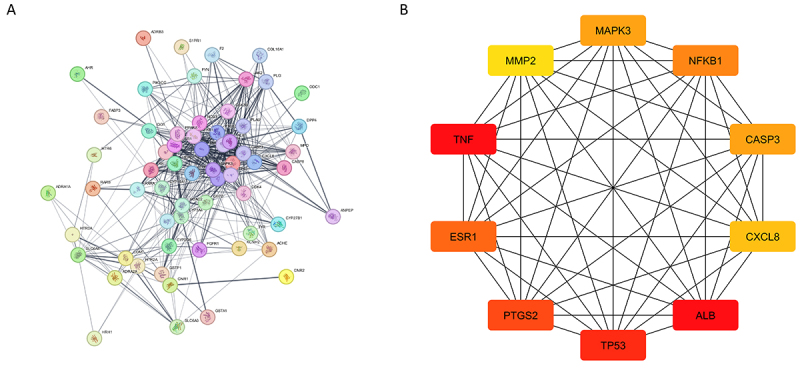
Protein-protein interaction (PPI) network and hub genes. **A**, The PPI network exhibited significant interactions. **B**, Top 10 hub genes ranked by their connectivity within the network.

The PPI network analysis (Figure 2A) revealed a highly interconnected network consisting of 63 nodes and 467 edges, which was significantly denser than expected (expected edges=184; PPI enrichment P-value<1.0e-16), with an average node degree of 14.8 and a high local clustering coefficient (0.648), indicating strong functional associations among proteins. Top hub genes identified in the network (Figure 2B) included *TNF*, *ESR1*, *MMP2*, *MAPK3*, *NFKB1*, *CASP3*, *CXCL8*, *PTGS2*, *TP53*, and *ALB*, suggesting their central roles in the network's biological processes due to their high connectivity.

### Gene ontology and pathway enrichment analysis

**Figure 3:**
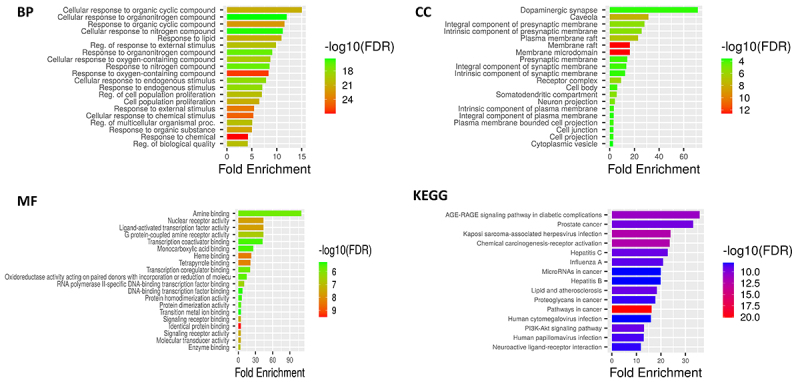
Molecular mechanisms of p-cymene-mediated protection against ethanol-induced umbilical cord-derived mesenchymal stem cells toxicity. Gene ontology and pathway enrichment analyses revealed biological processes (BP) related to chemical response and cell proliferation, cellular components (CC) associated with neuronal and membrane structures, molecular functions (MF) including transcription factor and oxidoreductase activities, and Kyoto Encyclopedia of Genes and Genomes (KEGG) pathways involved in oxidative stress response and cell survival. Bar lengths represent -log10 false discovery rate (FDR) values.

The gene ontology and pathway enrichment analyses provide detailed insights into the molecular mechanisms underlying P-cymene's protective effects against ethanol-induced toxicity in UC-MSCs (Figure 3). The biological process analysis revealed significant enrichment in cellular responses to chemical stimuli (FDR<1e-18), particularly involving organic cyclic and nitrogen compounds, suggesting P-cymene's role in mitigating ethanol's disruptive effects on cellular metabolism and proliferation. Cellular component analysis demonstrated strong enrichment in neuronal structures, including dopaminergic synapses (highest fold enrichment) and presynaptic membranes (false discovery rate (FDR)<1e-12), indicating P-cymene's potential to preserve synaptic integrity and membrane organization against ethanol-induced neurotoxicity. Molecular function analysis highlighted P-cymene's involvement in critical protective mechanisms through ligand-activated transcription factor activity (FDR<1e-9), oxidoreductase activity, and heme binding, which are essential for detoxification and oxidative stress response. KEGG pathway analysis further supported these findings, showing significant enrichment in the AGE-RAGE signaling pathway (FDR<1e-20), known to be involved in ethanol-induced oxidative damage, as well as in PI3K-Akt signaling and neuroactive ligand-receptor interactions, suggesting P-cymene's role in promoting cell survival and maintaining neuronal function. Additional enrichment in cancer-related pathways, such as chemical carcinogenesis and prostate cancer, implies p-cymene may counteract ethanol's genotoxic effects. Together, these results comprehensively demonstrate that p-cymene likely protects UC-MSCs through multiple coordinated mechanisms: 1) reducing oxidative stress via enhanced oxidoreductase activity and heme binding, 2) preserving cellular and synaptic membrane integrity, 3) activating critical cell survival pathways, and 4) counteracting ethanol-induced metabolic and genetic damage, providing a molecular foundation for its observed cytoprotective effects against ethanol toxicity (Figure 3).

### Molecular interactions of p-cymene with TNF-α, PTGS2, NF-κB, and MMP2

**Figure 4:**
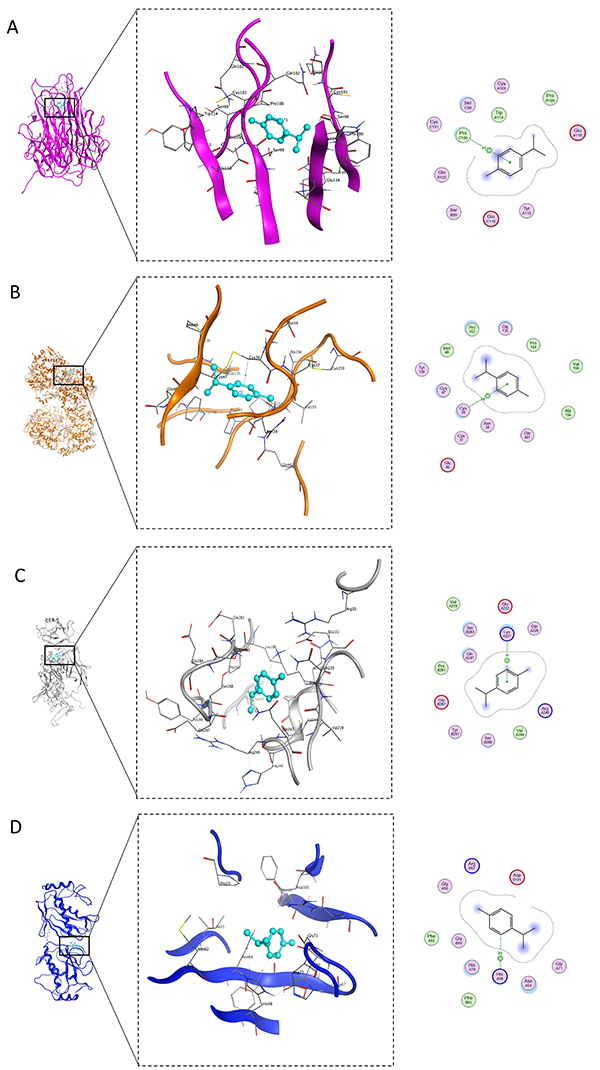
Molecular interactions of p-cymene with key targets. **A**, Binding pose with tumor necrosis factor (TNF)-α; **B**, Interaction with prostaglandin-endoperoxide synthase 2 (PTGS2); **C**, nuclear factor (NF)-κB complex; **D**, matrix metalloproteinase (MMP)2 binding site.

The molecular docking analysis revealed that p-cymene exhibited significant binding affinities with key molecular targets involved in ethanol-induced toxicity, demonstrating interaction energies ranging from -4.31 to -5.21 kcal/mol. p-cymene formed a pi-H interaction with PRO100 of TNF-α (S Score: -4.73 kcal/mol; distance: 4.15 Å), potentially modulating its inflammatory activity, while its interaction with CYS36 of PTGS2 (COX-2) through a similar pi-H bond (S Score: -4.66 kcal/mol; distance: 4.39 Å) suggested inhibition of prostaglandin synthesis. Notably, p-cymene showed the strongest binding to NF-κB (S Score: -5.21 kcal/mol) via a pi-cation interaction with LYS221 (distance: 3.94 Å), likely interfering with its DNA-binding capacity and subsequent pro-inflammatory signaling. Additionally, p-cymene's H-pi interaction with HIS98 of MMP2 (S Score: -4.31 kcal/mol; distance: 4.21 Å) indicated potential inhibition of extracellular matrix degradation. These specific interactions with TNF-α, PTGS2, NF-κB, and MMP2 at distances ranging from 3.94-4.39 Å, mediated through pi-H, pi-cation, and H-pi bonds, provided a structural basis for p-cymene's multi-target protective effects against ethanol-induced cellular damage, encompassing anti-inflammatory, antioxidant, and tissue-preserving mechanisms (Table 1 and Figure 4).

**Table 1:** Binding interactions and energetics of p-cymene with inflammatory and tissue remodeling proteins.

Compound	S Score (kcal/mol)	RMSD (Å)	Atom of compounds	Atom of receptors	Residue of receptor	Type of interactions	Distance (Å)	E (kcal/mol)
TNF-α (PDB ID 1TNF)								
p-Cymene	-4.7306	1.5050	6-ring	CA	PRO 100 (C)	pi-H	4.15	-0.5
NF-κB (PDB ID 1IKN)								
p-Cymene	-5.2078	0.7556	6-ring	NZ	LYS 221 (A)	pi-cation	3.94	-0.5
MMP2 (PDB ID 7XJ0)								
p-Cymene	-4.3054	0.6545	C4	5-ring	HIS 98 (A)	H-pi	4.21	-0.6

RMSD (Å): root mean square deviation in Angstrom; CA: alpha carbon; NZ: nitrogen atom in zeta position; LYS: lysine; HIS: histidine; PRO: proline; TNF-α: tumor necrosis factor-alpha; PDB: protein data bank; NF-κB: nuclear factor kappa-light-chain-enhancer of activated B cells; MMP2: matrix metallopeptidase 2.

### Morphology of UC-MSCs

**Figure 5:**
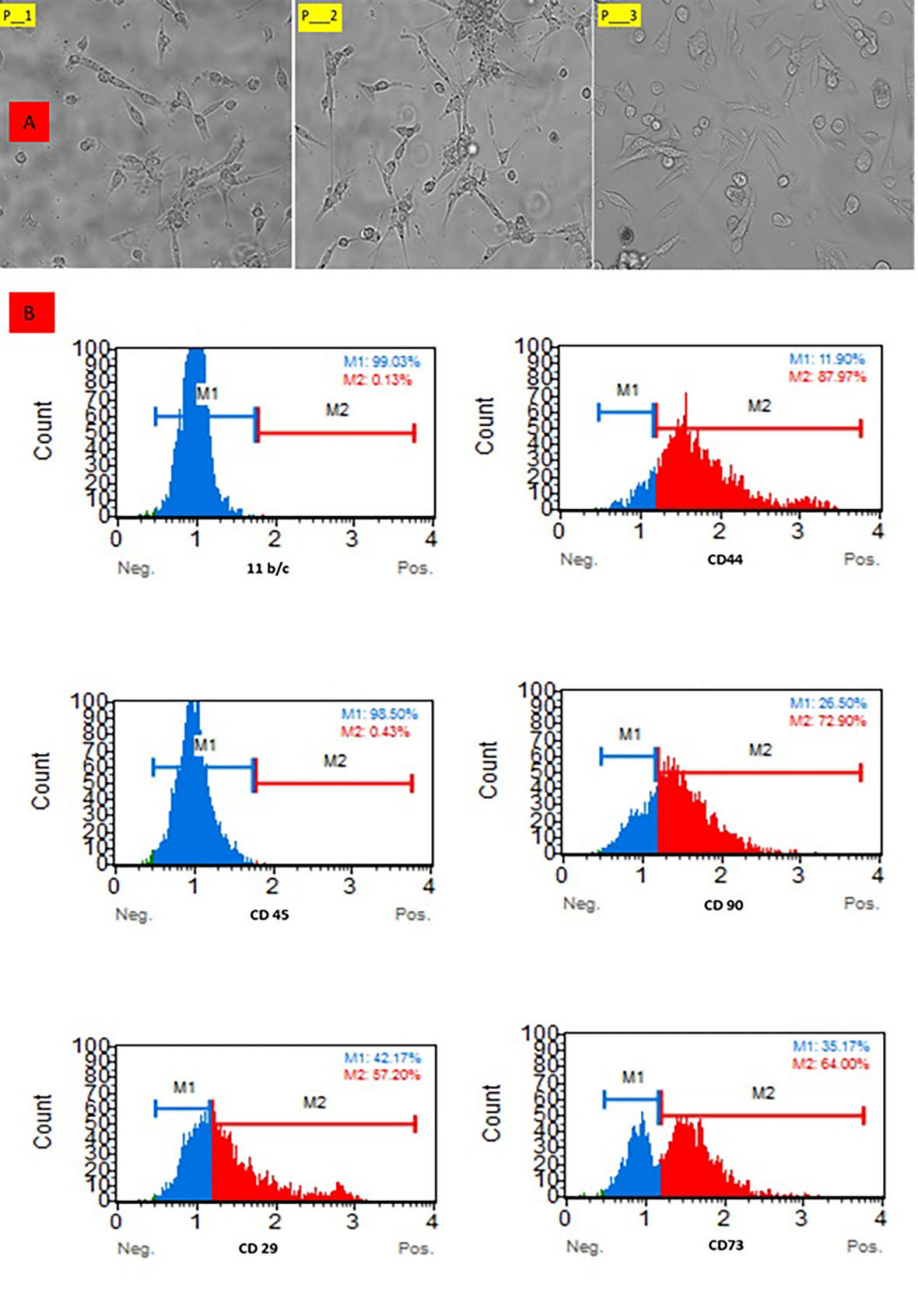
**A**, Morphological analysis of umbilical cord-derived mesenchymal stem cells at P1, P2, and P3 stages, showing more mature cells at P3. **B**, Flow cytometry analysis of mesenchymal stem cells showing negative expression for 11b/c and CD45, and positive expression for CD29, CD44, CD90, and CD73.

UC-MSCs exhibited a characteristic fibroblast-like morphology when cultured under standard conditions. Cells were spindle-shaped, adhered to the plastic surface, and formed monolayer colonies with a whirlpool-like arrangement upon reaching confluence. The morphology remained consistent across successive passages, indicating stable growth and self-renewal potential as shown in Figure 5A.

#### Surface marker characterization

Flow cytometry analysis was performed to confirm the immunophenotypic profile of UC-MSCs. The cells showed strong positive expression of UC-MSC-associated surface markers, including CD29, CD90, CD73, and CD44, while hematopoietic and myeloid lineage markers such as 11b/c and CD45 were absent. This expression pattern confirms the mesenchymal origin of the isolated cells and is consistent with the minimal criteria defined by the International Society for Cellular Therapy (ISCT) for UC-MSC characterization as shown in Figure 5B.

### Cell viability assay

**Figure 6:**
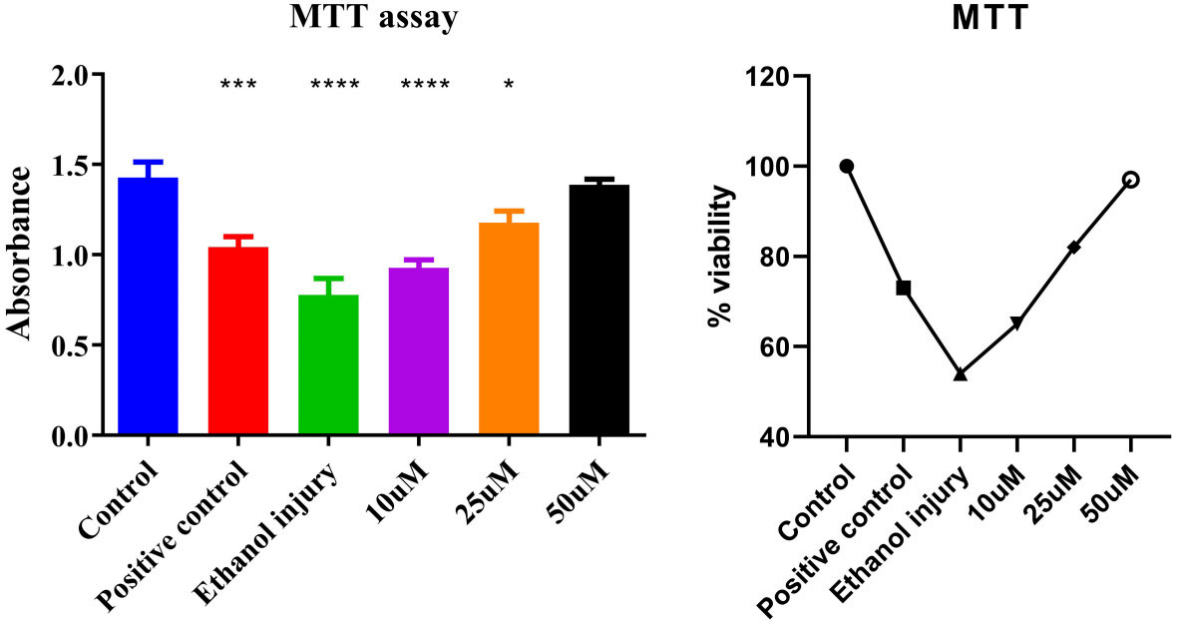
. Cytotoxicity analysis using MTT assay showing absorbance and percent viability graphs of the untreated (Control) group, Positive control group, Ethanol injury group, and groups treated with p-cymene at 10, 25, and 50 µM. Data are reported as means and SD. *P<0.05, ***P<0.001, and ****P<0.0001 compared to Control. ANOVA followed by Tukey's *post hoc* test.

There was a significant difference between control and treatment groups at lower concentrations of p-cymene, while higher concentrations maintained the cell viability in a concentration-dependent manner (10, 25, 50 µM) as shown in Figure 6.

### Detection of living and dead cells

**Figure 7:**
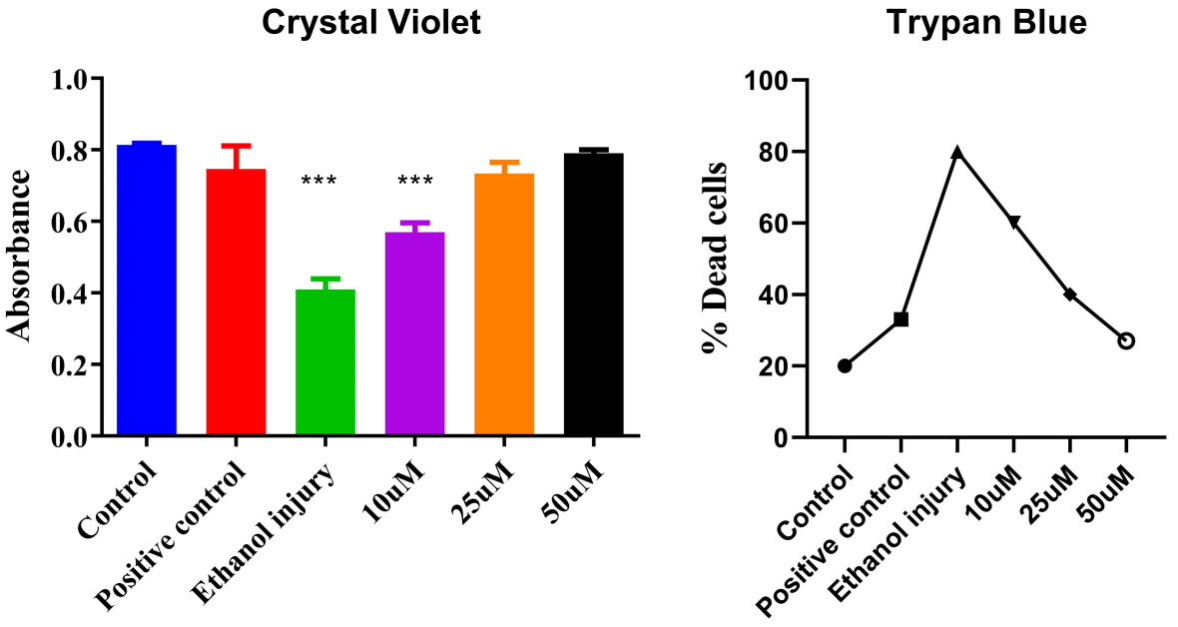
Crystal violet (live cell detection) and trypan blue (dead cell analysis) absorbance and dead cells graphs of the untreated group (Control), Positive control group, Ethanol injury group, and groups treated with p-cymene at 10, 25, and 50 µM. Data are reported as means and SD. ***P<0.001 compared to Control. ANOVA followed by Tukey's *post hoc* test.

The results of crystal violet staining indicated a concentration-dependent improvement in cell viability following p-cymene treatment after ethanol-induced injury. A greater number of live cells were observed in the groups treated with increasing concentrations of p-cymene (10, 25, and 50 µM) compared to the ethanol-injured group. Trypan blue was used to count dead cells. The UC-MSCs treated with ethanol showed a considerably higher number of blue-colored cells, while p-cymene had less blue colored cells in a concentration-dependent manner, suggesting very few dead cells in 50 µM, as shown in Figure 7.

### Inflammation, apoptosis, angiogenesis, and injury

**Figure 8:**
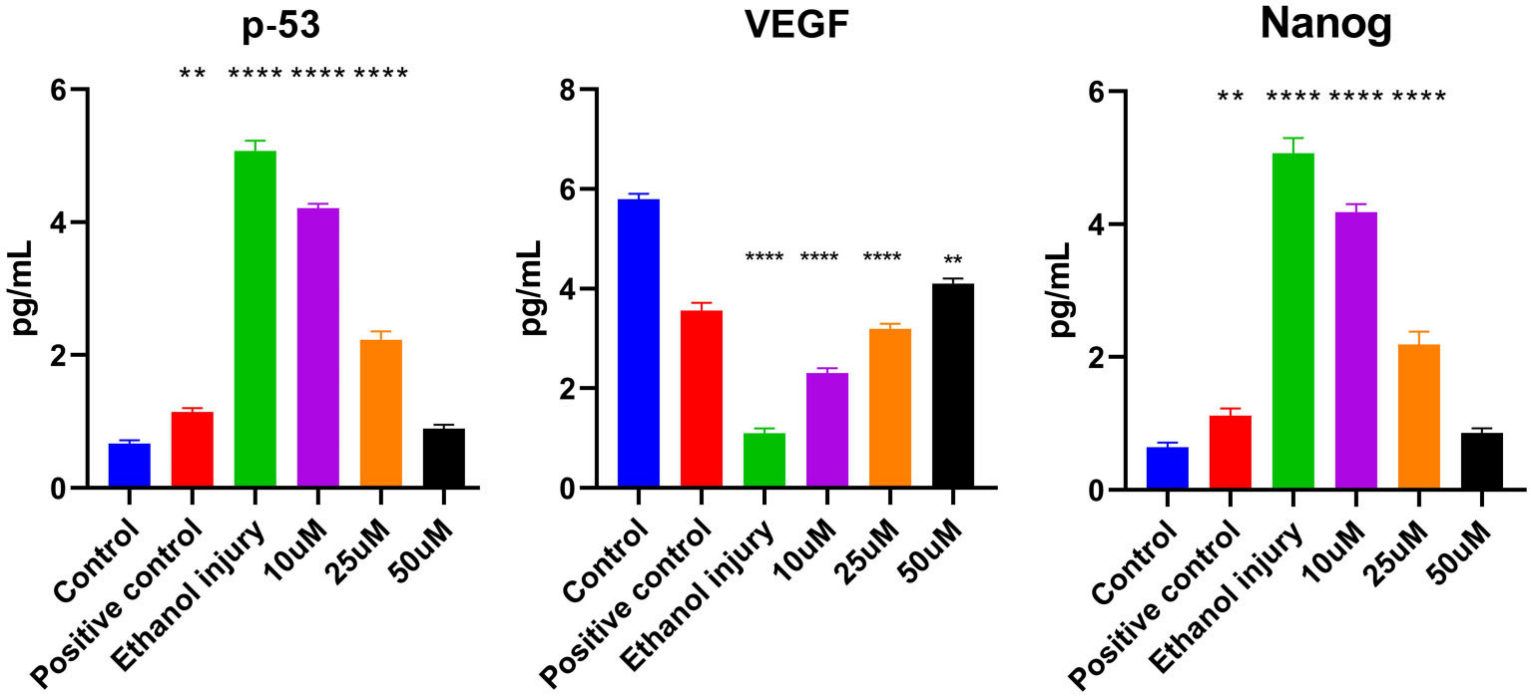
ELISA results of p-53, vascular endothelial growth factor (VEGF), and Nanog (*in vitro*) of the untreated group (control), positive control group, ethanol injury group, and groups treated with p-cymene at 10, 25, and 50 µM. Data are reported as means and SD. **P<0.01 and ****P<0.0001 compared to control. ANOVA followed by Tukey's *post hoc* test.

Protein expression of the apoptotic marker p53 was reduced in a concentration-dependent fashion upon treatment with p-cymene, suggesting its potential protective role against ethanol-induced cellular damage, as shown in Figure 8. The VEGF level in the control group was significantly decreased following ethanol-induced injury, indicating severe impairment in angiogenesis. Treatment with 10 µM p-cymene led to a partial recovery of VEGF expression. A further elevation was observed in the 25-µM treatment group. Notably, VEGF levels in the 50-µM treatment group were nearly restored to control levels, suggesting that p-cymene promotes angiogenesis and tissue repair as shown in Figure 8.

Conversely, Nanog levels, which were elevated as a response to ethanol-induced damage, showed a concentration-dependent reduction with p-cymene treatment. In the control group, Nanog levels significantly increased after ethanol exposure, highlighting the extent of cellular damage. Treatment with 10 µM p-cymene reduced Nanog levels, while a more substantial decline was observed in the 25-µM treatment group. At 50 µM, Nanog levels were nearly normalized to the control group levels, further supporting the protective effects of p-cymene against ethanol-induced cellular stress (Figure 8).

**Figure 9:**
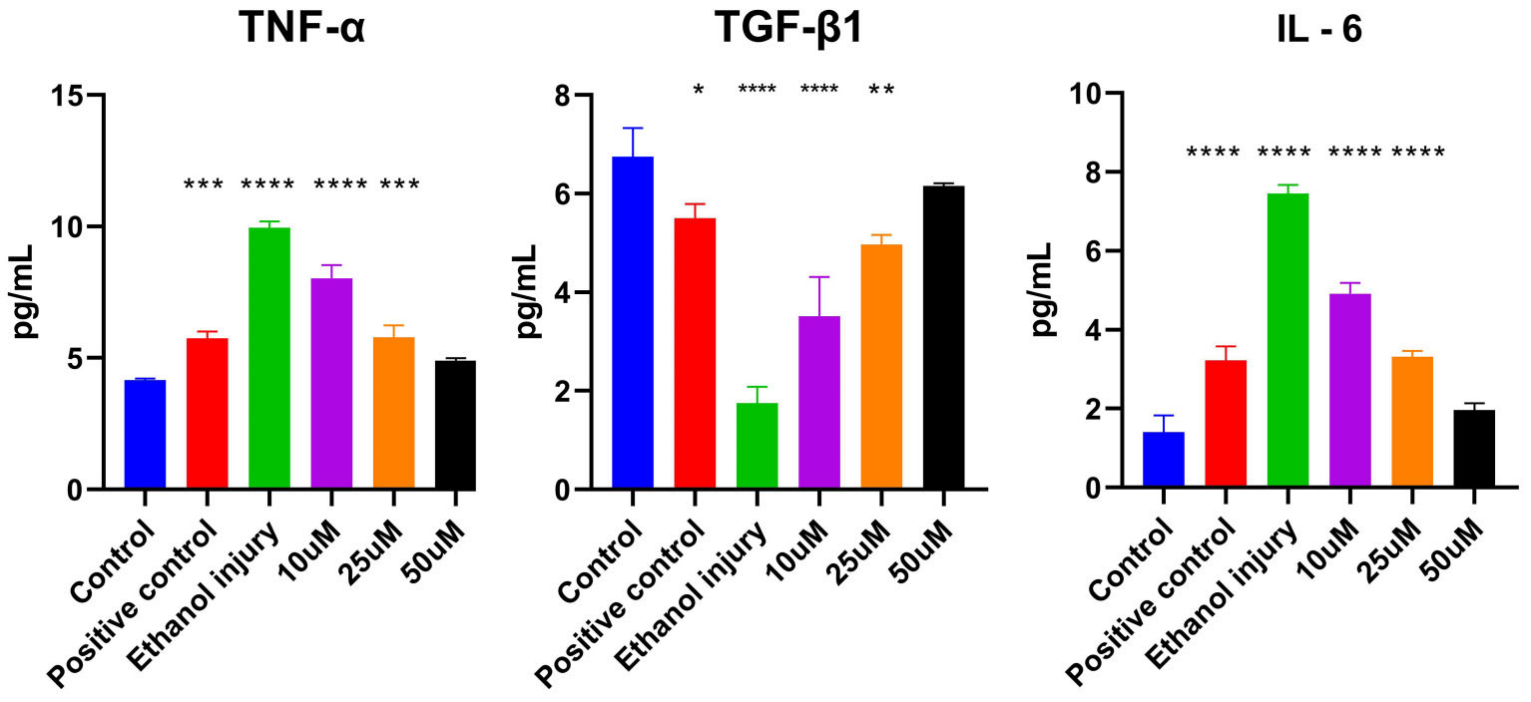
ELISA results of tumor necrosis factor (TNF)-alpha, transforming growth factor (TGF)-beta, and interleukin (IL)-6 (*in vitro*) of the untreated group (control), Positive control group, Ethanol injury group, and groups treated with p-cymene at 10, 25, and 50 µM. Data are reported as means and SD. *P<0.05, **P<0.01, ***P<0.001, and ****P<0.0001 compared to control. ANOVA followed by Tukey's *post hoc* test.

ELISA results showed a concentration-dependent reduction in the pro-inflammatory cytokines TNF-α and IL-6 in the p-cymene treatment groups. Conversely, TGF-β1 levels were significantly elevated in a concentration-dependent manner following treatment with p-cymene at 10, 25, and 50 µM (Figure 9).

### Antioxidant potential of p-cymene

**Figure 10:**
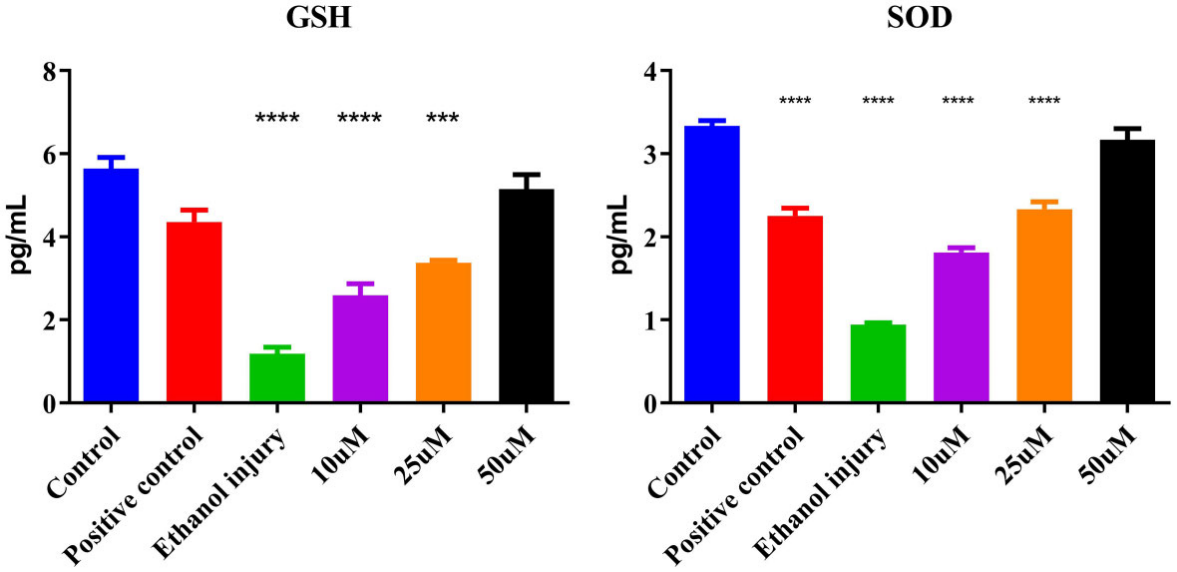
Glutathione (GSH) and superoxide dismutase (SOD) activity (*in vitro*) of the untreated group (Control), Positive control group, Ethanol injury group, and groups treated with p-cymene at 10, 25, and 50 µM. Data are reported as means and SD. *P<0.05, **P<0.01, ***P<0.001 compared to Control. ANOVA followed by Tukey's *post hoc* test.

The ethanol injury group showed a significantly lower mean GSH value compared to the control group, indicating a decrease in levels due to ethanol-induced injury. Treatment with p-cymene at concentrations of 10, 25, and 50 µM/mL partially restored GSH levels, as indicated by the significantly higher mean values compared to the ethanol injury group. Furthermore, a 50-µM/mL concentration had more potential for injury reversal compared with the positive control group (Figure 10).

The ethanol injury group showed a significantly lower SOD mean value compared to the control group, indicating a decrease in activity due to ethanol-induced injury. Treatment with p-cymene at concentrations of 50, 25, and 10 µM/mL resulted in increased mean activities. Furthermore, 50 µM/mL concentration had more potential for injury reversal compared with the positive control as shown in Figure 10.

## Discussion

The current study demonstrated that p-cymene exerted a protective role against ethanol-induced cytotoxicity in human stem cells, as evidenced by both computational and experimental approaches. Network pharmacology and docking analyses revealed strong interactions between p-cymene and key targets such as TNF-α and NF-κB, highlighting its potential to modulate inflammatory pathways. These *in silico* predictions were further validated *in vitro*, where p-cymene treatment improved cell viability, reduced oxidative stress markers, and restored angiogenic and reparative cytokines in a concentration-dependent manner. Collectively, these findings suggest that p-cymene not only mitigates ethanol-induced cellular injury but also promotes conditions favorable for stem cell survival and regeneration.

The initial phase of our study employed network pharmacology to identify key targets and understand the interactions between p-cymene and ethanol-induced toxicity. Utilizing tools such as Swiss Target Prediction and Pharm Mapper, we identified overlapping targets, which were then analyzed through PPI networks. The PPI analysis revealed a highly interconnected network, underscoring the complex biological pathways involved in ethanol toxicity and the potential mechanisms through which p-cymene exerts its protective effects. The identification of hub genes, including *TNF*, *PTGS2*, and *NF-κB*, indicated critical regulatory roles in inflammation and cellular stress responses, aligning with existing literature on ethanol-induced damage. Our identification of hub targets including *TNF-α* and *NF-κB* is consistent with earlier reports where p-cymene suppressed *NF-κB* and MAPK activation, reducing TNF-α and IL-6 (28).

Molecular docking studies provided further insights into the binding interactions between p-cymene and key target proteins. The docking results demonstrated significant binding affinities, with p-cymene forming stabilizing interactions with TNF-α and NF-κB. These interactions suggest that p-cymene may modulate inflammatory signaling pathways, thereby reducing the pro-inflammatory response associated with ethanol exposure. The findings from both network pharmacology and docking analyses support the hypothesis that p-cymene can effectively intervene in the pathways leading to ethanol-induced cytotoxicity.

The cytotoxicity of ethanol was assessed through the MTT assay (29,30), which confirmed significant reductions in cell viability following ethanol exposure. Our results indicated a concentration-dependent increase in cell viability with p-cymene treatment, particularly at concentrations of 25 and 50 µM. The near-complete restoration of cell viability at the highest concentration underscores p-cymene's protective capacity against ethanol-induced damage. This finding is consistent with previous studies highlighting the antioxidant effects of p-cymene, suggesting that its mechanisms may involve scavenging free radicals and reducing oxidative stress (31).

Crystal violet staining further corroborated these results, showing increased cell survival rates correlating with higher concentrations of p-cymene. This assay specifically quantifies viable cells by detecting intact nuclei, reinforcing the notion that p-cymene effectively preserves cell integrity in the presence of ethanol. The reproducibility of these results across different assays strengthens the case for p-cymene as a viable protective agent.

The trypan blue assay, which selectively stains dead cells, revealed a significant decrease in blue-stained cells with increasing concentrations of p-cymene. This confirms the cytoprotective role of p-cymene, as lower concentrations of p-cymene showed partial protection, while the highest concentration significantly reduced cell death. These findings are consistent with our hypothesis that p-cymene can mitigate ethanol-induced cytotoxicity through various protective mechanisms (12).

The ELISA analysis in our study assessed several key biomarkers, including TNF-α, IL-6, TGF-β1, p53, VEGF, and Nanog, each of which plays a crucial role in inflammation, apoptosis, and tissue repair processes. Understanding the significance of these markers and how p-cymene affects their expression provides insight into the therapeutic potential of p-cymene in mitigating ethanol-induced damage (32).

TNF-α and IL-6 are cytokines primarily produced by macrophages, playing a pivotal role in systemic inflammation. Their elevated levels are indicative of an inflammatory response, particularly in the context of ethanol exposure, which can exacerbate tissue damage (32,33). Our results demonstrated that p-cymene treatment led to a notable decrease in TNF-α and IL-6 levels, supporting its role as an anti-inflammatory agent. TGF-β1 is a multifunctional cytokine that plays a critical role in tissue regeneration and wound healing. It promotes cell proliferation, differentiation, and extracellular matrix production (34). Interestingly, our findings showed that treatment with p-cymene significantly increased TGF-β1 levels in a concentration-dependent manner. This elevation suggests that p-cymene not only mitigates the inflammatory response but also enhances the reparative processes necessary for tissue recovery following ethanol-induced injury. The dual effect of p-cymene in reducing pro-inflammatory cytokines while increasing TGF-β1 highlights its potential as a therapeutic agent in regenerative medicine.

The p53 protein is a key regulator of the cell cycle and is often referred to as the “guardian of the genome” due to its role in preventing genomic instability. Elevated p53 levels following ethanol exposure indicate cellular stress and apoptosis (35). Our results revealed that p-cymene treatment led to a significant reduction in p53 expression, nearly normalizing it to baseline levels. This suggests that p-cymene has a protective effect against ethanol-induced injury, enhancing cell survival by modulating the apoptotic pathways.

VEGF levels were assessed to explore the possible influence of p-cymene on growth factor modulation during wound healing. VEGF is a key regulator of endothelial cell proliferation, migration, and new vessel formation, and its upregulation is typically associated with enhanced tissue repair and angiogenic activity. Although our study did not directly evaluate angiogenesis through histological or imaging analyses, the observed increase in VEGF concentration in p-cymene-treated groups suggests that the compound may indirectly support vascular repair mechanisms, which aligns with a previous study (36). Treatment with p-cymene resulted in a concentration-dependent recovery of VEGF expression, suggesting that it not only protects against cytotoxicity but also enhances angiogenic processes. This restoration of VEGF levels further supports the potential of p-cymene in promoting tissue regeneration following ethanol damage.

Nanog levels serve as a marker for cellular stress and damage (37). In our study, we observed a significant increase in Nanog levels following ethanol exposure, which was progressively reduced with p-cymene treatment. This decrease in Nanog levels indicates that p-cymene effectively mitigates the cellular stress response induced by ethanol, suggesting its protective role in preserving cellular integrity.

Overall, our findings illustrate that p-cymene exerted a multifaceted protective effect against ethanol-induced cellular damage by modulating key inflammatory and apoptotic pathways. The significant reductions in pro-inflammatory cytokines (TNF-α and IL-6), the increase in the reparative cytokine (TGF-β1), the decrease in the apoptotic marker (p53), the restoration of the angiogenic factor (VEGF), and the reduction of the stress marker (Nanog) collectively justify the potential of p-cymene as a therapeutic agent in conditions associated with alcohol-induced toxicity. These results underscore the need for further exploration of p-cymene in clinical settings to fully realize its therapeutic benefits in regenerative medicine.

### Study limitations

A limitation of the present study is that the protective effects of p-cymene were not validated in an animal model, which restricts direct translation of the findings to *in vivo* conditions. Future studies will focus on confirming these results in relevant animal models and exploring the underlying molecular mechanisms in greater depth.

## Data Availability

All the data have been incorporated into the manuscript.
